# Prohibitin-induced obesity leads to anovulation and polycystic ovary in mice

**DOI:** 10.1242/bio.023416

**Published:** 2017-04-21

**Authors:** Sudharsana Rao Ande, Khanh Hoa Nguyen, Yang Xin Zi Xu, Suresh Mishra

**Affiliations:** 1 Department of Internal Medicine, University of Manitoba, Winnipeg, R3E 3P4Canada; 2 Department of Physiology & Pathophysiology, University of Manitoba, Winnipeg, R3E 0J9Canada

**Keywords:** Transgenic models, Periovarian adipose tissue, Cystic ovary, Female infertility

## Abstract

Polycystic ovary syndrome (PCOS) is a prevalent endocrine disorder and the most common cause of female infertility. However, its etiology and underlying mechanisms remain unclear. Here we report that a transgenic obese mouse (Mito-Ob) developed by overexpressing prohibitin in adipocytes develops polycystic ovaries. Initially, the female Mito-Ob mice were equally fertile to their wild-type littermates. The Mito-Ob mice began to gain weight after puberty, became significantly obese between 3-6 months of age, and ∼25% of them had become infertile by 9 months of age. Despite obesity, female Mito-Ob mice maintained glucose homeostasis and insulin sensitivity similar to their wild-type littermates. Mito-Ob mice showed morphologically distinct polycystic ovaries and elevated estradiol, but normal testosterone and insulin levels. Histological analysis of the ovaries showed signs of impaired follicular dynamics, such as preantral follicular arrest and reduced number, or absence, of corpus luteum. The ovaries of the infertile Mito-Ob mice were closely surrounded by periovarian adipose tissue, suggesting a potential role in anovulation. Collectively, these data suggest that elevated estradiol and obesity per se might lead to anovulation and polycystic ovaries independent of hyperinsulinemia and hyperandrogenism. As obesity often coexists with other abnormalities known to be involved in the development of PCOS such as insulin resistance, compensatory hyperinsulinemia and hyperandrogenism, the precise role of these factors in PCOS remains unclear. Mito-Ob mice provide an opportunity to study the effects of obesity on anovulation and ovarian cyst formation independent of the major drivers of obesity-linked PCOS.

## Abstract (precis)

**Summary:** Obesity-related overgrowth of periovarian adipose tissue and elevated estradiol levels cause anovulation and ovarian cyst formation in mice, independent of hyperinsulinemia and hyperandrogenism.

## INTRODUCTION

The incidence rate of infertility is on the rise worldwide. Ovulatory defects and unexplained causes account for more than 50% of infertile etiologies (Talmor and Dunphy, 2014). It is postulated that a significant proportion of these are either directly or indirectly related to obesity. The prevalence of overweight and obese men and women has surpassed 50% in some developed countries (Talmor and Dunphy, 2014). Along with the increase in the prevalence of overweight and obesity in women, there has been an increase in anovulatory infertility (Giviziez et al., 2016). Androulakis et al. (2014) reported that visceral adiposity index is related to the severity of anovulation and other clinical features in women with polycystic ovaries. An increase in subcutaneous abdominal fat has also been associated with anovulation in women with obesity (Kuchenbecker et al., 2010). It is believed that anovulatory infertility accounts for 25-50% of causes of female infertility (Weiss and Clapauch, 2014). One of the main causes of anovulatory infertility is polycystic ovaries (Ben-Shlomo et al., 2008; Messinis et al., 2015). The ability of the adipose tissue to accumulate sex hormones and to metabolize and interconvert them through local enzymatic activities can significantly affect the functional status of the reproductive axis (Giviziez et al., 2016). In addition, several substances produced by adipose tissue, including leptin, adiponectin and resistin, might play a role in the pathophysiology of polycystic ovaries. Infertility resulting from ovulatory disorders is a growing concern, because the prevalence of overweight and obesity in women of reproductive age has increased considerably during the past 30 years (Best and Bhattacharya, 2015). A systematic analysis of literature on overweight, obesity and ovulatory disorders has found that there is consensus regarding the negative impact of obesity on anovulatory infertility (Giviziez et al., 2016). However, how it affects ovulatory function is still under investigation.

Anatomically, ovary and visceral adipose tissue share the same abdominal compartment in the body and are present in close proximity. Therefore, the possibility exists that in the case of increased visceral obesity, adipose tissue might grow over ovaries and create a physical barrier for ovulation from the ovarian surface. Theoretically, such a condition could lead to the development of polycystic ovaries as it is widely agreed that ovarian cysts develop as a consequence of anovulation (Bozdag et al., 2016). In addition, an increase in serum estradiol levels due to obesity might alter the feedback regulation at the hypothalamus-pituitary axis and contribute to ovarian cyst formation. Because obesity often coexists with other abnormalities such as hyperinsulinemia, insulin resistance and altered sex steroid levels, which are known to be associated with PCOS (Franks et al., 1999; van Houten et al., 2012), it is difficult to pinpoint the local effects of periovarian adipose tissue on the development of polycystic ovaries. Similarly, it is challenging to discern the development of PCOS due to primary changes in the ovary under such conditions. Recently, we developed a transgenic obese mouse model, Mito-Ob, by overexpressing prohibitin in adipocytes from the adipocyte protein-2 (*aP2*) gene promoter (Ande et al., 2014). Mito-Ob mice develop obesity during the post-pubertal period in a sex-neutral manner, but obesity-associated insulin resistance and compensatory hyperinsulinemia occur only in males (Ande et al., 2014). Female Mito-Ob mice, despite being significantly obese, have normal glucose homeostasis and insulin sensitivity similar to their wild-type littermates. Thus, female Mito-Ob mice have provided an opportunity to investigate the role of obesity-related local changes in anovulation and, consequently, on ovarian cyst formation, independent of factors that generally coexist with obesity and are linked with PCOS. Here we report that Mito-Ob mice spontaneously develop anovulatory polycystic ovaries independent of insulin resistance, compensatory hyperinsulinemia and hyperandrogenism.

## RESULTS

### Obesity in female Mito-Ob mice leads to reduced fecundity, ovarian cyst formation and infertility

**Fig. 1.**
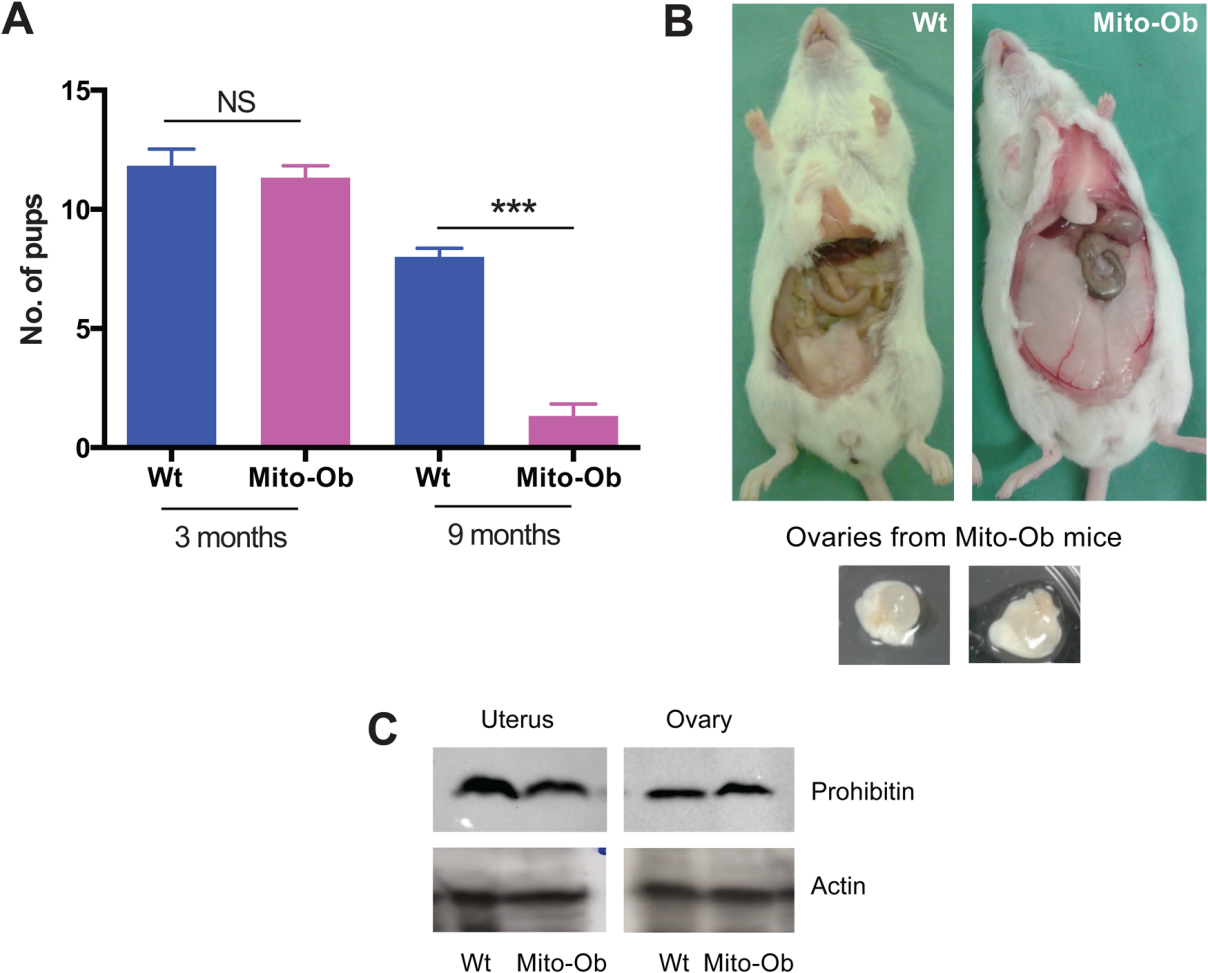
**Mito-Ob mice display obesity, polycystic ovaries and reduced fecundity.** (A) Histograms showing litter sizes in Mito-Ob and wild-type (Wt) mice at 3 and 9 months of age. ***P<0.001 by Student’s *t*-test between Mito-Ob and male Wt mice. Data are presented as mean±s.e.m (*n*=6 mice/group). NS, not significant. (B) Representative photographs showing visceral obesity in a female Mito-Ob mouse at 6 months of age. A female Wt mouse is shown as a control (upper left). Ovaries dissected from Mito-Ob mice are also shown (lower panels). (C) Representative immunoblots showing prohibitin levels in the uterus and ovary of Mito-Ob and Wt mice. Beta-actin blots are shown as a loading control (*n*=3).

Recently, we reported a novel transgenic obese mouse model (Mito-Ob) developed by overexpressing prohibitin in adipocytes (Ande et al., 2014). At 6 months of age, Mito-Ob mice had significantly higher body weight as reflected by increased visceral and subcutaneous adipose tissue mass with hypertrophied adipocytes (Ande et al., 2014). During follow-up studies on Mito-Ob mice, we noticed a significant reduction in fecundity with aging in comparison with wild-type mice, and the female Mito-Ob mice were infertile by 9 months of age (Fig. 1A). Observations of reduced fecundity and subsequent loss of fertility in Mito-Ob mice prompted us to investigate the ovarian phenotype of Mito-Ob mice. At 9 months of age, ovaries from ∼25% of Mito-Ob mice were significantly enlarged, owing to the formation of morphologically apparent ovarian cysts filled with serous fluid (Fig. 1B), which was not observed in the ovaries from wild-type mice. The ovarian cysts in Mito-Ob mice often developed bilaterally.

To know if the *aP2* gene promoter is active in the female reproductive tract and ovary in Mito-Ob mice, which might have contributed to ovarian dysregulation, we determined prohibitin levels using western immunoblotting with anti-prohibitin antibody (Ande et al., 2014). We found no significant difference in prohibitin levels in the uterus and ovary of Mito-Ob mice compared with wild-type control mice (Fig. 1C). These data suggest that the observed ovarian phenotype in Mito-Ob mice is not caused by prohibitin overexpression in the female reproductive tract.

**Fig. 2.**
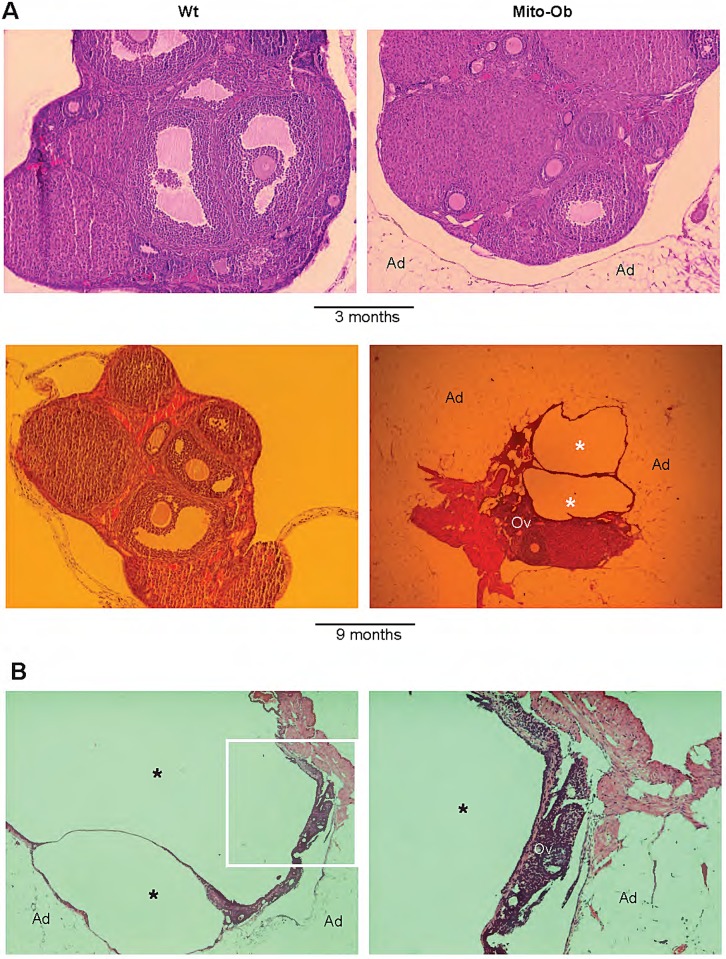
**Histological staining showing changes in ovarian structure in Mito-Ob mice and close association between ovaries and periovarian adipose tissue.** Representative photomicrographs showing H&E-stained ovarian sections from (A) Wt and Mito-Ob mice at 3 and 9 months of age, (B) an infertile Mito-Ob mouse at 9 months of age. The right panel shows a magnified view of the boxed area in the left panel (*n*=6 mice/group). Ad, adipose tissue; Ov, ovary; asterisk, ovarian cyst.

**Fig. 3.**
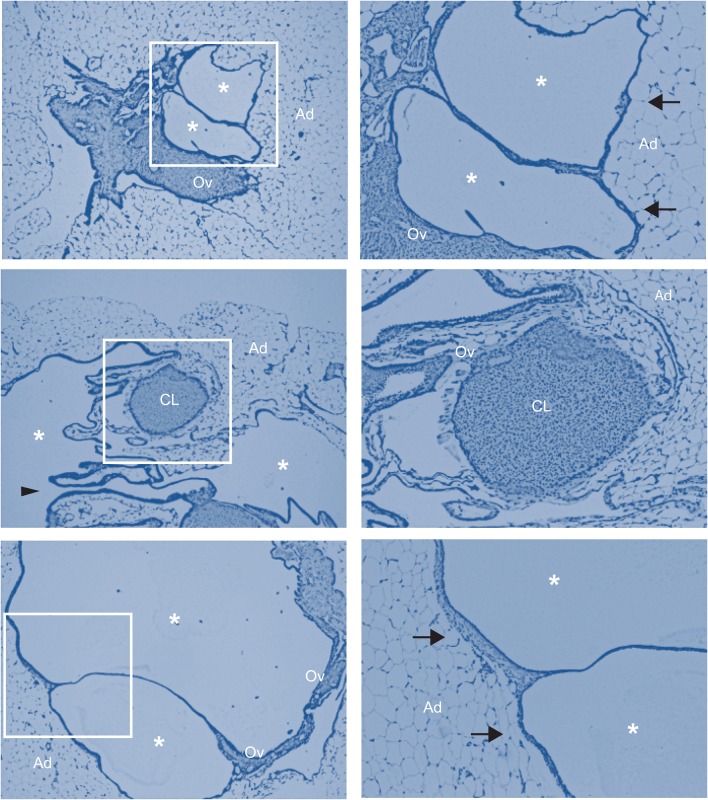
**Histological staining of ovaries from infertile Mito-Ob mice showing close association between ovaries and periovarian adipose tissue.** Representative photomicrographs of ovaries from 9-month-old Mito-Ob mice showing ovarian cysts at different stages of their development. A blue filter was used to improve visualization of the adipose tissue. The right panels show magnified views of the boxed areas in the left panels. Arrowhead indicates highly extended ovarian cyst wall; arrow indicates close association between the ovarian surface and periovarian adipose tissue (*n*=6). Ad, adipose tissue; CL, corpus luteum; Ov, ovary; asterisk, ovarian cyst.

Histological analysis of the ovaries from Mito-Ob mice confirmed ovarian cyst formation (Fig. 2A,B). Furthermore, ovaries were surrounded by overgrowth of adipose tissue and periovarian adipose tissue closely associated with the ovarian epithelial surface (Figs 2A,B and 3). The arrest of follicular development and a reduction in corpus luteum number, or their absence, was apparent in the ovaries of Mito-Ob mice (Figs 2A,B and 3). With increasing ovarian cyst size, the ovarian tissue mass was found to be pushed to one side and compressed, as a result of substantial enlargement of the ovarian cysts from one side and overgrowth of periovarian adipose tissue from the other side (Figs 2B and 3).

### Obesity does not affect glucose homeostasis and insulin sensitivity during infertility development in female Mito-Ob mice

**Fig. 4.**
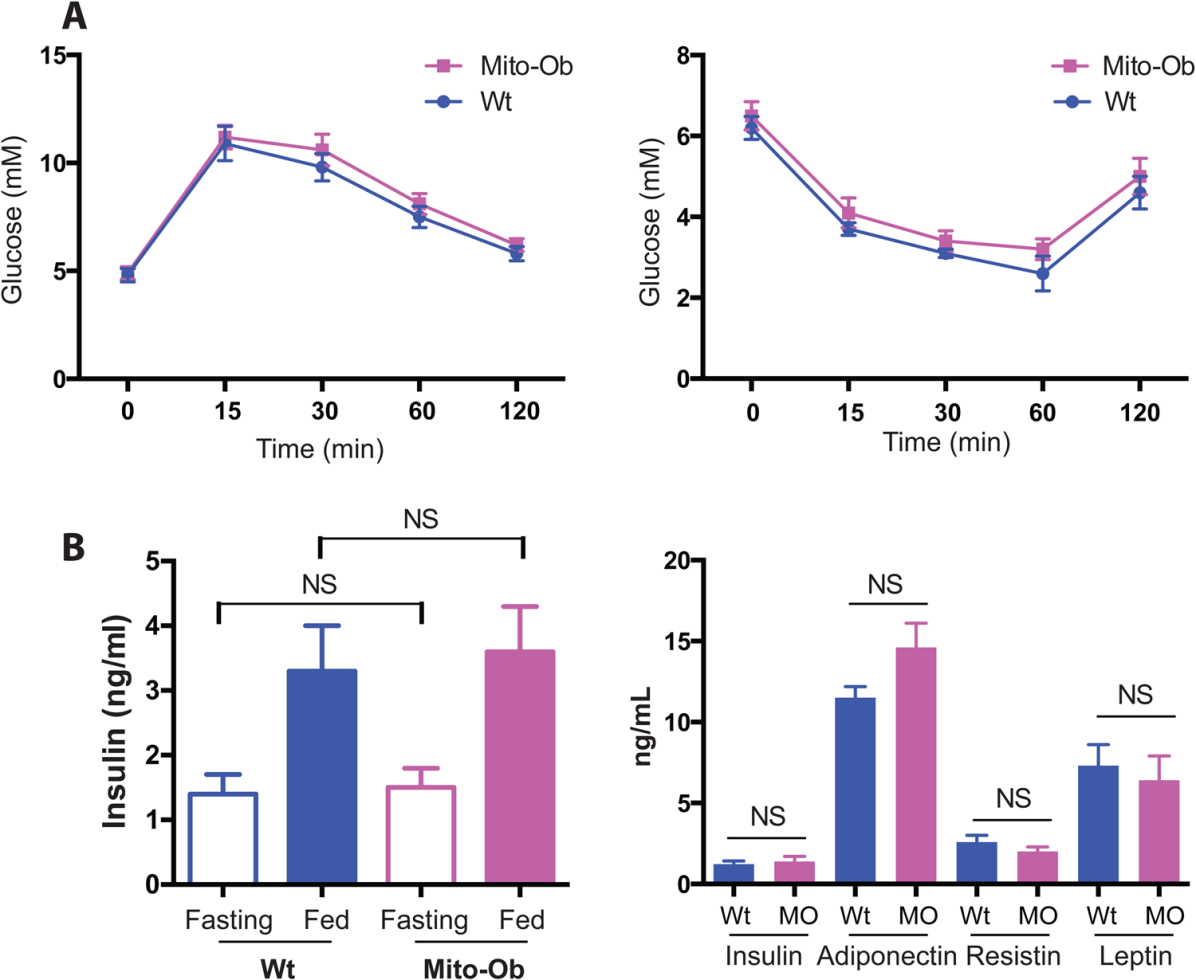
**Metabolic phenotype and hormone levels in female Mito-Ob mice during ovarian cyst development.** (A) Line graphs showing glucose tolerance test (GTT, left) and insulin tolerance test (ITT, right) results in 9-month-old Mito-Ob mice compared with Wt mice. Data are presented as mean± s.e.m. (*n*=6 mice/group). (B) Histograms showing fasting and fed insulin levels (left) and different hormone levels (right) in Mito-Ob mice and Wt mice. Data are presented as mean±s.e.m. (*n*=6 mice/group). NS, not significant by Student’s *t*-test between Mito-Ob and Wt mice. MO, Mito-Ob.

Recently, we reported that obesity in female Mito-Ob mice has no effect on glucose homeostasis and insulin sensitivity at 6 months of age (Ande et al., 2014). To avoid comparison with the previous study and to determine metabolic status during obesity-related infertility, we measured glucose homeostasis in Mito-Ob mice at 9 months of age. We found no differences in glucose and insulin tolerance between the Mito-Ob mice and age-matched wild-type mice (Fig. 4A, left and right). Consistent with normal glucose homeostasis and insulin sensitivity, we found no significant differences in fasting and fed serum insulin levels between the Mito-Ob and wild-type mice (Fig. 4B, left). Similarly, leptin and resistin levels were not significantly different between the Mito-Ob and wild-type mice, whereas adiponectin levels were higher in the Mito-Ob mice compared with in the wild-type mice (Fig. 4B, right). Collectively, these data suggest that glucose homeostasis and insulin sensitivity remain normal during obesity-related ovarian dysfunction and infertility in Mito-Ob mice.

### Luteinizing hormone (LH) and testosterone levels remain normal during ovarian cyst development in Mito-Ob mice

**Fig. 5.**
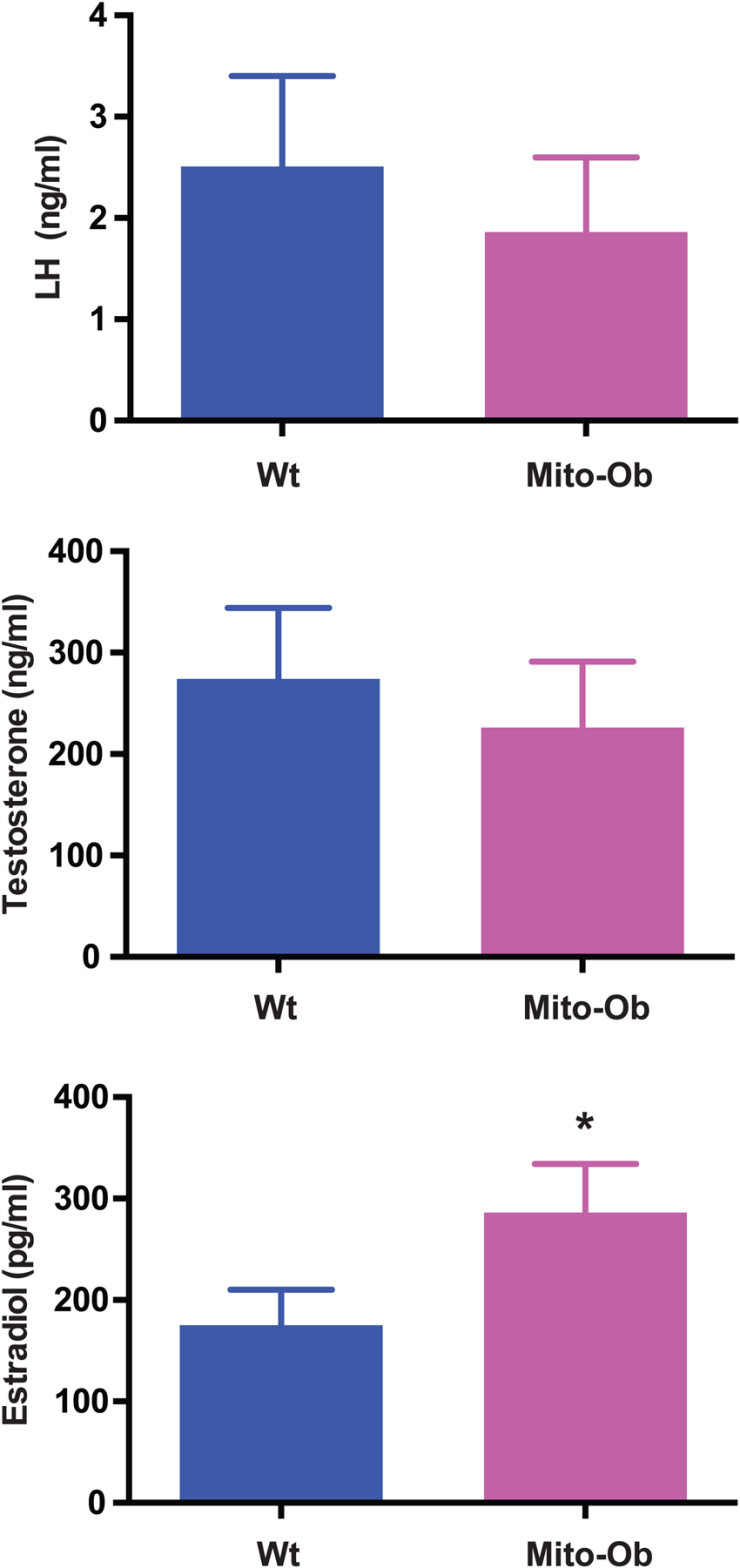
**Serum hormone levels in Mito-Ob mice.** Histograms showing LH (upper), testosterone (middle) and estradiol (lower) levels in female Mito-Ob mice at 9 months. Data are presented as mean±s.e.m. (*n*=6 mice/group). NS, not significant, *P<0.05, by Student’s *t*-test between Mito-Ob and Wt mice.

Hyperandrogenism and hyperinsulinemia are considered important contributory factors in the development of PCOS in women (Restuccia et al., 2012). Our findings of normal insulin levels and insulin sensitivity, along with anovulation and polycystic ovaries, in Mito-Ob mice prompted us to measure LH and testosterone levels in female Mito-Ob mice. We observed no significant differences in serum LH and testosterone levels between the Mito-Ob and wild-type mice (Fig. 5), although the levels of both hormones were lower in the Mito-Ob mice than in wild-type mice. Taken together, these data suggest that obesity-related ovarian dysregulation and infertility in Mito-Ob mice develop independent of hyperinsulinemia and high androgen levels.

### Obesity leads to increased estradiol levels in Mito-Ob mice

Visceral adipose tissue is known to express aromatase (also known as estrogen synthase), which aromatizes testosterone to estradiol (Zeng et al., 2014). In rodent models, exogenous administration of estradiol leads to the development of polycystic ovary (Chapman et al., 2009). Thus, there exists the possibility that an increase in estradiol levels due to obesity has a role in ovarian cyst development in Mito-Ob mice. We therefore aimed to determine serum estradiol levels in our Mito-Ob mice. Serum estradiol was significantly elevated in the Mito-Ob mice compared with in wild-type mice (Fig. 5), indicating a potential role for elevated serum estradiol in anovulation and ovarian cyst development in Mito-Ob mice.

## DISCUSSION

Here we report a novel and spontaneous transgenic mouse model of obesity-associated anovulation and polycystic ovary independent of insulin resistance. The ovarian phenotype in Mito-Ob mice provides evidence that obesity and elevated estradiol levels are sufficient to cause anovulation and ovarian cyst formation. Furthermore, polycystic ovary might arise due to primary defect in the ovary and overgrowth of periovarian adipose tissue, independent of factors normally implicated in its development such as increased androgens and insulin levels. Altered follicular dynamics, as observed in the ovaries of Mito-Ob mice, is consistent with the notion that ovulation is a crucial event in the normal functioning of the follicular cycle, and that anovulation negatively affects follicular dynamics and leads to preantral follicular arrest and cyst formation (Devin et al., 2007; Walters et al., 2012). In this context, it should be noted that plasminogen activator inhibitor-1 transgenic mice, which have an anovulatory phenotype due to ovarian changes, also develop ovarian cysts (Devin et al., 2007). Another related example is estradiol-induced PCOS models (Chapman et al., 2009). Exposure to estradiol leads to morphological features of anovulation and polycystic ovaries similar to those of PCOS patients (Chapman et al., 2009). Collectively, these studies suggest that factors that lead to anovulation have the potential to contribute to the development of PCOS. Thus, ovarian cyst formation in Mito-Ob mice might be caused by the physical barrier formed by overgrowth of periovarian adipose tissue around the ovary as found in our histological analysis, or by high estradiol levels as a result of obesity, or a combination of both. It is possible that altered microenvironment at the interface of the ovary and the periovarian adipose tissue (MIOP), especially when the ovary is completely surrounded by adipose tissue, negatively affects the follicular dynamics within the ovary. Furthermore, enrichment of various factors in the MIOP that are released from ovary and periovarian adipose tissue might facilitate interconnection between the ovarian surface and periovarian adipose tissue. Thus, a combination of local changes in follicular dynamics and overgrowth of periovarian adipose tissue could lead to anovulatory infertility and polycystic ovary in mice, independent of hyperinsulinemia and hyperandrogenism.

Hyperandrogenism is the most consistent feature of women with PCOS (Abbott et al., 2002). However, it is not clear whether hyperandrogenism is a cause, or a consequence, of PCOS, although a number of androgen-induced animal models of PCOS have been developed, suggesting that androgens are an important factor in the development of PCOS (van Houten and Visser, 2014; Walters et al., 2012). In this context, it is important to note that such effects of androgens are restricted to specific stages in the developmental process (van Houten and Visser, 2014). As the source and the timing of such effects of hyperandrogenism in the development of PCOS in women are not clear, the possibility of androgen-independent etiology of human PCOS cannot be ruled out. Furthermore, high androgen levels generally seen in PCOS patients might be secondary to the development of PCOS. For example, hyperinsulinemia might contribute to hyperandrogenism through its action on theca cells in PCOS patients. The polycystic ovarian phenotype of Mito-Ob mice opens up an opportunity to explore androgen-independent development of polycystic ovary.

In addition to Mito-Ob mice, we have recently reported a mutant Mito-Ob mouse expressing a phospho mutant (Y114F) form of prohibitin in a similar manner (Ande et al., 2016). The mutant Mito-Ob mice share the obese and metabolic phenotype of Mito-Ob mice, but develop lymph node tumors only in males, suggesting a protective role of estrogen in obesity-related metabolic dysregulation and tumor development (Ande et al., 2016). As speculated, ovariectomy in mutant-Mito-Ob mice was found to impair metabolic dysregulation and lymph node tumor development (Ande et al., 2016), confirming a protective role of estrogen in these mice. Furthermore, mutant Mito-Ob mice display anovulation and polycystic ovary similar to female Mito-Ob mice (S.M., unpublished data). The development of polycystic ovary in mutant-Mito-Ob mice further supports the findings from Mito-Ob mice that overgrowth of periovarian adipose tissue and elevated estradiol can cause ovarian dysregulation and infertility including polycystic ovary.

It is anticipated that the spontaneous models of obesity-related anovulation and polycystic ovary described here will be valuable to explore the underlying mechanisms involved in obesity-related anovulation and ovarian disorders. Thus, Mito-Ob mice will be helpful to discern the relative contribution of different factors generally co-existing in obesity-related ovarian disorders.

## MATERIALS AND METHODS

### Generation of transgenic Mito-Ob mice

The generation of transgenic Mito-Ob mice overexpressing prohibitin in adipocytes has been described elsewhere (Ande et al., 2014). All animal works reported here were performed as per the study protocol approved by the Institutional Animal Care and Use Committee at the University of Manitoba, Canada. For comparisons between Mito-Ob and wild-type mice, male Mito-Ob mice were bred with wild-type CD-1 female mice to generate hemizygous Mito-Ob mice and wild-type mice from the same litter. Thus, wild-type control mice were of an identical genetic background to the transgenic Mito-Ob mice. The Mito-Ob transgenic mice were identified by genotyping the tail DNA using the polymerase chain reaction (PCR) with the following primers: forward primer: 5′-GCAGCCCGGGGGATCCACTA-3′ and 5′-GCACACGCTCATCAAAGTCCTCTCCGATGCTG-3′. The Mito-Ob and wild-type mice were housed in small groups with 12-h dark-light cycles and with access to food and water *ad libitum*.

### Western immunoblotting

The uterus and ovary lysates from Mito-Ob and wild-type mice containing equal amounts of proteins (15 mg/lane) were separated by sodium dodecyl sulfate polyacrylamide gel electrophoresis (SDS-PAGE) and subsequently analyzed by immunoblotting using anti-prohibitin antibody (Ande et al. 2014).

### Histology

Ovaries from 3- to 9-month-old Mito-Ob and wild-type mice were fixed overnight in 10% buffered formaldehyde and subsequently dehydrated in a graded series of ethanol, before being embedded in paraffin. Five-micrometer ovarian sections were cut and processed for Hematoxylin and Eosin (H&E) staining (Lei et al., 2001; Nguyen et al., 2011).

### Measurement of serum hormones and adipokine

Serum insulin and adipokine levels were measured using mouse Bio-Plex Pro™ Diabetes Assay Panels and Bio-Plex 200™ multiplex suspension array systems (Bio-Rad) as per the manufacturer's protocols (Ande et al., 2014, 2016). Levels of LH (USBiological, Swampscott, MA, USA) and sex steroid hormones (Cayman Chemical, Ann Arbor, MI, USA) were measured using commercially available kits as per the protocols provided by the manufacturer.

### Glucose and insulin tolerance tests

Glucose and insulin tolerance tests in 9-month-old Mito-Ob mice and their corresponding wild-type littermates were performed as per the standard procedures described in our previous publications (Nguyen et al., 2011; Ande et al., 2014).

### Statistical analysis

All statistical analyses were performed using GraphPad Prism 6 (La Jolla, CA, USA). Experimental results are shown as mean±s.e.m. Two-tailed Student's unpaired *t*-tests were performed to compare Mito-Ob and wild-type littermates, unless indicated otherwise. *P*<0.05 was considered significantly different.
